# Contraceptive methods and use by women aged 35 and over: A qualitative study of perspectives

**DOI:** 10.1186/1472-6874-11-5

**Published:** 2011-02-16

**Authors:** Emily M Godfrey, Nancy P Chin, Stephen L Fielding, Kevin Fiscella, Ann Dozier

**Affiliations:** 1 Department of Family Medicine, University of Illinois College of Medicine and Community Health Sciences, University of Illinois School of Public Health, Chicago, IL, USA; 2 Department of Community and Preventive Medicine, University of Rochester School of Medicine and Dentistry, Rochester, NY, USA; 3 Department of Clinical & Social Sciences in Psychology, University of Rochester, Rochester, NY, USA; 4 Department of Family Medicine, University of Rochester School of Medicine and Dentistry, Rochester, NY, USA; 5 Department of Community and Preventive Medicine, University of Rochester School of Medicine and Dentistry, Rochester, NY, USA

## Abstract

**Background:**

More than 30% of the pregnancies in women aged 35 and over are unintended. This paper compares perceptions about contraceptive methods and use among women with and without an unintended pregnancy after turning age 35.

**Methods:**

Semi-structured, in-depth interviews were conducted with 17 women. They were all 35 to 49 years old, regularly menstruating, sexually active, not sterilized, not desiring a pregnancy in the near future, and at least 3 months postpartum. We purposely sampled for women who had had at least one unintended pregnancy after age 35 (n = 9) and women who did not (n = 8). We assessed partnership, views of pregnancy and motherhood, desired lifestyle, perceived advantages and disadvantages of using and obtaining currently available well-known reversible contraceptives in the U.S. ''We also assessed contraceptive methods used at any time during their reproductive years, including current method use and, if appropriate, circumstances surrounding an unintended pregnancy after age 35.'' Each interview was taped and transcribed verbatim. Data were analyzed using Grounded Theory. Analysis focused on partnership, views of pregnancy, motherhood, desired lifestyle and perceived advantages and disadvantages of various reversible contraceptive methods.

**Results:**

The women without an unintended pregnancy after age 35 were more likely to (1) use contraceptive methods that helped treat a medical condition, (2) consider pregnancy as dangerous, or (3) express concerns about the responsibilities of motherhood. The women who experienced an unintended pregnancy after age 35 were more likely to (1) report unstable partnerships, (2) perceive themselves at lower risk of pregnancy, or (3) report past experiences with unwanted contraceptive side effects. There was a greater likelihood a woman would choose a contraceptive method if it was perceived as easy to use, accessible, affordable and had minimal side effects.

**Conclusions:**

Women's perspective on contraceptive use after age 35 varies. Public health messages and health providers' care can help women in this age group by reviewing their fertility risks, as well as all contraceptive methods and their associated side effects. The impact of such interventions on unintended pregnancy rates in this age group should be tested in other areas of evidence-based medicine.

## Background

Although the number of women deferring pregnancy to later reproductive years is increasing, many pregnancies are still unintended[1]. In 2001 (the latest year in which data are available), 29% of pregnancies were unintended among women aged 35 to 39, and 38% were unintended for women over age 40[2]. Unintended pregnancy in older women is problematic because inherent obstetrical risks increase during the late reproductive years. U.S. women aged 35-39 have a pregnancy-related mortality ratio twice that of women aged 25-29 (deaths per 100,000 live births = 21.3 vs. 9.1), and women aged 40 and over have a nearly five times greater ratio (45.5 vs. 9.1)[3]. Additionally, women aged 35 and over have a higher risk of stillbirth than women in their 20 s and early 30 s, even when risk factors are taken into account (O.R. = 1.3 for women aged 35-39, 1.7 for women aged 40 and older)[4].

Contraception decreases the probability of unintended pregnancy and associated risks. Women over age 35 are more than 3 times more likely to forgo contraception use than women aged 20-24[5]. Contraceptive choices of women over 35 have changed throughout the last decade. Although sterilization is still the most commonly chosen contraception for women over age 35, more in recent years are using reversible hormonal methods or withdrawal and rhythm methods[6]. Nearly 20% of women aged 40-44 and 15% women aged 35-39 report no contraception use[6].

A plethora of review articles promoting the use of various reversible contraceptive methods are available to help direct health care providers who care for women in their later reproductive years,[7-10] but factors that underlie older women's beliefs about, choices of, and use of various reversible contraceptive methods are not well understood. Although many consider women in their later reproductive years as less fecund,[11] reasons for high rates of contraceptive non-use are not entirely clear. In a behavioral-focused review paper, Ketting suggests that the factors that influence contraception use by older women may be different than for younger women. Women in their late reproductive years have greater concern for health, changing sexual relationships, doubts about their risk of pregnancy, or uncertainty about their role in society[12].

In this paper, we compare the perceptions about contraceptive methods and use among women with and without an unintended pregnancy after age 35. We examine the extent to which an older woman's perceived risk of unintended pregnancy, partner status, personal experience with contraception, and perceived advantages and disadvantages of using and obtaining currently available well-known contraceptive methods affects contraceptive choice and use in U.S. women aged 35 and over.

## Methods

We conducted semi-structured, in-depth interviews with 17 women aged 35 and over in the Greater Rochester area (New York) from July through November 2002. Prior research evaluating optimal sample size for an in-depth interview study suggests 15 to 20 respondents is sufficient for recurring themes to emerge from the participants' responses[13]. The University of Rochester Institutional Review Board (IRB) approved the study protocol. Inclusion criteria included women aged 35 to 49, regularly menstruating, sexually active, not sterilized, not desiring a pregnancy in the near future, and at least 3 months postpartum[14]. Given the exploratory nature of this study and the evidence that suggests that race and ethnicity, education and income affect contraception use,[5] we specifically attempted to include women from various racial, ethnic, and socioeconomic backgrounds by collecting information about total household income (including government assistance), occupation and educational level.

We recruited participants through several mechanisms, including flyers posted in workplaces, family planning clinics, hospitals and women's shelters and word-of-mouth referrals from neighborhood contacts and other study participants. Women who telephoned seeking further information were screened to determine if they met the study criteria. During the screening call, we obtained obstetrical histories to include women who had had at least one unintended pregnancy after age 35 and women who had not had an unintended pregnancy after age 35.

If the woman qualified for the study, an interview was arranged at a time and place of the participant's choosing. The interviewer made a reminder call the day before the interview to confirm the appointment. The interviewer obtained informed written consent in person before starting the interview. Each interview was face-to-face, ranging from 45 to 90 minutes, audio-recorded, and transcribed verbatim. The principal investigator (PI) conducted 12 interviews, and two research assistants conducted three each (six total) for 18 overall. The research assistants underwent interview training by observing two interviews by the PI, performing one mock interview and then being observed by the PI for one interview. The PI also reviewed the transcripts of the six interviews conducted by the research assistants for quality assurance.

The semistructured interview guide contained 15 open-ended questions. The questions addressed partnership, views of pregnancy and motherhood, desired lifestyle, perceived advantages and disadvantages of using and obtaining currently available well-known reversible contraceptives in the U.S., contraceptive methods used at any time during their reproductive years, including current method use, and, if appropriate, circumstances surrounding an unintended pregnancy after age 35. Each participant completed a demographic questionnaire that consisted of nine closed-ended questions (participant's age, residential zip code, payment method used to purchase birth control, ethnic and racial background, highest grade completed, marital status, occupation, and total family income, including funds from government assistance).

Data were analyzed using the Grounded Theory system developed by Strauss,[15] as well as the editing style proposed by Miller and Crabtree[16]. The editing style approach involves segmenting the data by identifying the information most pertinent to the research questions. Next, the research team blocked off lines of text that conveyed and supported the themes of the interview. To increase the reliability of the analysis, two team members re-read the lines of text within the context of the interview and coded them with words or phrases that represented the particular idea or concept mentioned by the respondent, using the "open code" technique proposed by Strauss[16,17]. Once the team agreed on each code, the PI entered the code into electronic format using Atlas.ti (Berlin, Germany). Comments within the software program were used to clarify the meaning of a code and to ensure consistency between the interviews. Some codes from earlier interviews were re-worded, particularly when later interviews clarified or provided new insights.

## Results

Eighteen women were interviewed. One woman had never been sexually active, despite stating so on the screening questionnaire and thus was excluded from the analyses. Nine women reported at least one unintended pregnancy after age 35, eight did not. The respondent characteristics are listed in Table 1. Most women with at least one unintended pregnancy after age 35 were currently using male condoms, whereas many women who did not have an unintended pregnancy after age 35 were currently using combined oral contraceptive pills. Only two respondents (both who had had an unintended pregnancy after age 35) were currently using methods considered "very effective" by the World Health Organization (intrauterine contraception [IUC] and contraceptive implant)[18].

**Table 1:** Participant Characteristics

Characteristic	Unintended pregnancy after age 35	No unintended pregnancy after age 35
	**(n = 9)**	**(n = 8)**

**Race/Ethnicity**		

African American	7	1

White	1	7

Latina	1	0

**Marital Status**		

Single/Never married	5	0

Married, living with husband	0	8

Divorced or separated	4	0

Unmarried, living with partner	0	0

**Highest Educational Level**		

Some high school	2	0

Completed high school	3	0

Some college	3	1

Completed college or higher	1	7

**Total Family Income Prior Year to Interview**		

Less than $10,000	4	1

$10,000 to $19,999	2	0

$20,000 to $29,999	0	1

$30,000 to $39,999	1	0

$40,000 to $49,999	0	0

$50,000 or greater	2	6

**Mean number of children**	2.8	2.0

**Attitudes toward future childbearing**		

Do not desire more children	4	6

Ambivalent	4	2

Desire more children	1	0

Effectiveness of Reversible Contraceptive Used Within Prior Week of Interview*		

Very Effective	2	0

Effective	2	6

Moderately Effective	5	2

Less Effective	0	0

*Contraceptive effectiveness is defined according to the *World Health Organization in Family Planning: A global handbook for providers*. Baltimore and Geneva: CCP and WHO, 2007, Appendix A, p. 319

Factors influencing contraception use, especially between many women who had an unintended pregnancy after age 35 versus those who did not, were varied. The women without an unintended pregnancy after age 35 were more likely to (1) use contraceptive methods that helped treat a medical condition, (2) consider pregnancy as dangerous, or (3) express concerns about the responsibilities of motherhood.

### Treatment of a Medical Condition

Respondents who reported using a contraceptive method to treat an existing medical condition, such as a gynecological disorder tended to report contraception adherence. I.T. is a 46-year-old woman without a prior history of unintended pregnancy after age 35. She is currently using the pill, which she described as helping her menopausal symptoms:

As I get older, I've learned that they're also an advantage because they're supposed to ease you into the menopausal stage relatively easily, so that you have less symptoms of menopause. It's supposed to ease hot flashes and so forth, so that's one of the reasons I'm staying on it.

I.T. reports she tried other birth control methods including male condoms and the diaphragm, but that she likes the pill the best because she learned from her doctor that it would ease her symptoms into menopause.

N.S., is a 48-year-old woman who had an unintended pregnancy at age 37 while using condoms inconsistently. She reports after her unintended pregnancy she received reassurance from her physician that the pill was safe and would help resolve her chronic menstrual disorder:

I have always had really horrible periods and was diagnosed with anovulation when I was in my... 20 s and so... for many years I was on the pill and then for many years I just had lots of break-through bleeding and lots of complications so I...went off [the pill] and had D&C's because of the anovulation. And so, I used other forms of birth control, like condoms and then in my late... 30's after my unplanned pregnancy, I got put back on the pill and I've been on the pill ever since. And that was really to control heavy bleeding. ... I mean that's been a God-send...

Although N.S. had an unintended pregnancy after age 35 while using condoms, she reports successful use of the oral contraceptive pill for more than a decade because of her understanding and first hand experience that the pill is treating her chronic medical condition of anovulatory bleeding.

### Dangers of Pregnancy

Respondents who perceived becoming pregnant as dangerous at their particular age, also expressed meticulous use with their contraception method. S.C. is a 40-year-old woman with two children and no history of prior unplanned pregnancy after age 35. She explains how she had had two previous difficult pregnancies and is currently struggling to find the right birth control method. S.C.'s mother was diagnosed with breast cancer after age 65, which prompted her to stop considering any contraceptive method containing estrogen. She chose to use the progesterone-only pill and struggles with the uncertainty of its lower efficacy rate. S.C.'s personal difficulties from her previous pregnancies motivate her to use contraception diligently. She explains that she missed a pill several months ago and was worried about possibly becoming pregnant when her period was late because "it's too much of a risk." She reflects about her close call with an unintended pregnancy:

I would have weighed everything against it, being older, difficult pregnancy, risks of something going wrong, disadvantages of problems on the family, in terms of it being hard on my husband, him having much more to do, it's very difficult for him. And it would be hard for my other two children. I would have taken a break from parenting them because I was flat out on the sofa [throughout my previous pregnancies].... So, our family is complete... I pretty much never miss a pill...

In recognizing that pregnancy itself is difficult and that it could impose health problems, S.C. pays close attention to taking her progesterone-only pill.

F.H., a 41-year-old woman who had an unintended pregnancy just before she turned 35, states she uses condoms with every act of intercourse. She was diagnosed with a clotting disorder during her last pregnancy and had to take medication postpartum:

I don't take any chances.... I know that it would be dangerous for me to have more [children] now because of this clotting disorder, so I don't really take any more chances. Not that I ever took too many anyway. I didn't take any except once and I got pregnant.

F.H. was impacted by her unintended pregnancy, which technically occurred just before she turned 35. Nonetheless, from that pregnancy she learned that pregnancy is more dangerous as she gets older, particularly with presence of a medical condition. Her expressed fear of developing health problems with a pregnancy appears to motivate F.H. to use condoms consistently.

### Concerns about the Responsibilities of Mothering

Respondents who expressed apprehensions about mothering tried to be conscientious about their contraception use. R.Z. is a 37-year-old, childless woman and no history of unintended pregnancy after age 35 who was recently married and desires children with her new husband. He, however, is satisfied with the two children he has from a previous marriage. Although ambivalent about pregnancy, R.Z.'s concerns about the responsibilities of motherhood and desire for leisure influence her contraception use:

I haven't had any children. We're in the process of trying to decide whether we want to have children now or not and I'm really, really on the fence. It's a hard decision to make. Had we been facing this choice even five years ago, I absolutely would have started a family. But now I'm thinking that by the time I would actually *get *pregnant and have the child, I'm looking at being 40 and 40 and [having] a toddler and I'm considering going to graduate school and there's just so many pieces in the decision that weren't as significant factors before. So now it's really a lifestyle decision. I would love to have a child. I would love to parent..., but I'm thinking towards retirement....

R.Z. seems to have considered her responsibilities in being a mother very seriously. She appears ambivalent about having a child in the context of other things she would to have in her life, such as being a graduate student.

The women who tended to avoid unintended pregnancy after age 35 talked about their adherence to contraception in the context of a medical condition, perceiving pregnancy as dangerous or expressing concerns about the responsibilities of motherhood. In contrast, the women who experienced an unintended pregnancy after age 35 were more likely to (1) perceive themselves at lower risk of pregnancy, (2) report past experiences with unwanted contraceptive side effects or (3) report tentative partnerships.

### Perceived Decreased Risk of Pregnancy

A number of the women who had an unintended pregnancy after age 35 did not perceive themselves as fertile and thus ceased using contraception. A 44-year-old woman named A.A. with 3 children did not desire more children. A.A. did not have a favorable view of birth control, and in particular, disliked the diaphragm because she felt it interfered with heightened sexual pleasure. A.A. wanted to forgo contraception and thought she could not get pregnant:

I thought that at my age that my fertility rate would be a lot lower. At 44, I'm going to be 45 in September. It's unbelievable. I figured, I'm not going to get pregnant. I just got my period. I know it's a safe time. Because I know ten days before your period and ten days after is a good time to know it's safe. I was wrong.

A.A. believed she was not as fertile given that she was in her mid-40 s. She also mentions using periodic abstinence, but appears to have knowledge gaps regarding correct timing of the "safe" period.

D.F., aged 40, talked about how she is using the "rhythm method" (or periodic abstinence) as her choice of birth control. When asked why, she stated:

I think it's a lot easier and I'm not taking medicine and putting things and chemicals into my system I really don't need. And plus they say when you get over 35, once you're over 40, your chances of getting pregnant decrease. It's not that you can't get pregnant, but the chances decrease. And I was taking birth control for so long, that I was just like, you know my body needs to rest. So I just stopped.

D.F. had two abortions after age 35. She said her unplanned pregnancies came from "laziness" and from "not taking a birth control method and umm, startin' em and stoppin' em." D.F. had used condoms, pills and depot medroxyprogesterone (DMPA) in the past, but was unfamiliar with some of the newer methods, such as intrauterine contraception (IUC), patch and a monthly injectable contraceptive (which was available in the U.S. at the time of this study). To D.F., she had tried the gamut of available birth control methods, and periodic abstinence seemed like the safest, most hassle-free choice, particularly in light of her perceived low risk of pregnancy.

### Unwanted Side Effects

Even when women were not seeking pregnancy, several respondents talked about the intolerable side effects that they experienced from birth control and as a consequence, stopped using them. J.K. was a 36-year-old woman who had had an unintended pregnancy less than one year prior to the interview. She talked about how she experienced bleeding after one injection of depot medroxyprogesterone (DMPA) given immediately postpartum: "I bled for almost two months....Oh my God. I mean constantly, nonstop I thought I was going to die. I mean, literally. I was wearing my baby's Pampers. ... [I]t was ridiculous, and it was scary."

J.K.'s negative experience with DMPA was a disappointment for her. She states that when she went back to her clinic to tell them about the bleeding, she was told the bleeding should stop and was not given any other options about what she could use. Although J.K. had already had several unintended pregnancies, the negative experience from the side effects of DMPA made her unwilling to continue the method. "It didn't do like they said," J.K. replies, and not knowing what other method to turn to, had another unplanned pregnancy soon after she discontinued this method.

### Partnership

Unstable partnerships tended to negatively influence contraception use. This finding has been noted in other research studies[19]. These women tended to view that being in love with their partners did not warrant contraceptive use, that the partnership alone justified the possibility of having another child. L.G. is a 43-year-old woman who had a recent unintended pregnancy. Prior to the unplanned pregnancy, L.G. and her husband were separated. They then got back together, and L.G. felt that contraception use was less important:

I don't know HOW I had him [my son]. I just thought, I had probably have another baby (laughs lightly). I was going with .., you know, me and my husband started going having in and out, in and out, and then get back and I just said, ooh, what the heck [to using contraception].

L.G. is not thinking about her concerns about the responsibilities of motherhood, but rather the possibility that pregnancy may solidify her relationship with her husband.

When talking about her unplanned pregnancy two years prior, A.A. reports a similar theme regarding her hope for a fortified relationship:

I never wanted Lisa. I didn't want to be pregnant. But I was happy I was pregnant because I was in love with the man I was with. Meaning I wanted it; however, I didn't want to do it alone. Meaning I had reservations. I did, I was so happy because I was so in love with the father, maybe he'll love me more that I have his child.

When asked if the love increased after the pregnancy and birth of "Lisa," A.A. responds:

No. It never happened that way.... My expectations of having this child were more on getting the man than having the child, for me. You know, I was hoping, I'll love it, and that he was going to love me more and I'm going to get him in the process. I really believed that. That was the first time I ever tricked... it wasn't trickery but it was 'I'm pregnant and boy...' I kind of had an advantage, a leverage, as they say, women do these things. I did it too. And it didn't work.... He didn't come around the way I was expecting.

Despite this disappointment and the lack of desire for more children, A.A. was unable to shift her thinking about contraceptive use: "I had two abortions since Lisa. And I had one prior to that. So what does that tell ya? That I say it, meaning it was all with the father. I mean when I'm with this man that I love, I'm somewhere else mentally."

M.G., a 35-year-old woman with three children who does not desire more children, explains her reaction to her last pregnancy, which occurred several months before the interview, as: "Oh Lord! not again." But when asked about the events that led up to this unplanned pregnancy, she says:

I really wasn't too big on birth control because I never really felt I needed birth control 'cause I was always with the same guy ... I wasn't worried about getting pregnant; it really didn't matter one way or another, you know, if I was pregnant.

Women who had at least one unintended pregnancy after age 35 tended to view contraception as a hassle, something to avoid if not completely necessary, whereas many women who had avoided an unintended pregnancy after age 35 viewed contraception as something that protected them from the potential health hazard of pregnancy, alleviated a medical problem, or allowed them to avoid the responsibilities of motherhood.

### Contraception Selection

Although some women were successful at using moderately effective contraceptive methods (such as condoms), the investigators asked the respondents about their perceived advantages or disadvantages of contraceptive methods they had used in the past, as well as those they had never used to better understand why more effective methods, such as intrauterine contraception (IUC) methods were not being used. Respondents framed their responses in the context of safety, efficacy, accessibility, ease or difficulty. Respondents appeared to have knowledge gaps with newer methods, such as the subdermal implant, monthly injectable, and contraceptive patch or ring because many had not used these methods, nor were familiar with them.

Perceptions that made respondents feel more favorable about selecting and using a method included spontaneity, ease, and accessibility.

### Spontaneity

Both women with and without an unintended pregnancy after age 35 felt favorable about a contraceptive method that did not interfere with lovemaking. For example, K.T., a 40-year-old woman without an unintended pregnancy after age 35, talks about an advantage of the pill:

You don't have to worry about putting any of that in prior. You can just be spontaneous. You don't have to worry about, 'oh wait just a second.' You know, we're in the heat of the moment. Let me run to the bathroom and put the diaphragm in. Or let me insert this vaginal contraceptive or something like that. You don't have to worry about that.

### Ease

Both women with and without an unintended pregnancy after age 35 felt favorable about easy and convenient methods and unfavorable about methods that were "technical" (such as the diaphragm) or involved "remembering" (such as the pill).

### Accessibility and Service Barriers

Accessibility appeared to influence use. Most women perceived over-the-counter methods as accessible and easy to get. An exception to this was the pill, which was considered convenient if medical providers gave a one-year prescription. B.W., aged 35 woman and a history of unintended pregnancy after age 35, talks about the convenience of the male condom:

They are easy to get, and they're free. If you choose to go the STD clinics or Planned Parenthood, they give you as many as you want. They are very easy. They have them in bathrooms, I understand... Very easy to obtain and reasonably priced.

Perceptions that tended to make respondents feel less favorable about selecting and using a method included being dangerous, costly and hard to obtain.

### Dangerous

Most women felt unfavorably about methods that were perceived as harmful. Although most women who had not had an unintended pregnancy after age 35 reported intrauterine contraception (IUC) or the intrauterine device (IUD) as effective, many believed it causes pelvic inflammatory disease (PID). Some women who had had an unintended pregnancy after age 35 perceived IUC as hazardous; it was "internal" and had the potential to migrate somewhere else in the body. E.M., aged 49 with four children and no history of unintended pregnancy after age 35, does not have personal experience with IUC, but relays stories from friends:

I've had friends that used the IUD and maybe it was because it was fairly new, but it got embedded and they had to have surgery to get it out, and it just seemed like not a good thing to me. I was scared of that.

I.T., a 46-year-old without a prior history of unintended pregnancy after age 35 who is using the pill because she understands it will ease her menopausal symptoms, states: "I've heard that there are complications from ... the IUD. I know someone who has a lot of problems with bleeding and other things. . .I guess it's improved over the years. Um, so I'm not real familiar with the newer versions, but back in my 20's I used to hear a lot of horror stories about it."

G.R., a 39-year-old with three children and a history of unintended pregnancy after age 35, explains her apprehensions with intrauterine contraception, stating she "wouldn't trust it." When asked why, G.R. states: "I don't know, it just don't look right. Something might happen...like it will travel. You see, I mean you can't keep something like that in place unless you inflate it, you know what I'm saying?"

Other respondents reported fears about chemically-based hormonal contraception as being potentially harmful. S.G., a 42-year-old woman without a history of unintended pregnancy after age 35, relates:

I would wonder what is this chemical, this drug or whatever that is, that is going into my body that is going to prevent me from getting pregnant that length of time. If it's three months, for three whole months what in the world is this that I'm putting in my body?

### Cost

Choosing a birth control method on the basis of cost was reported more often by those with no insurance or private insurance requiring co-pays than those with Medicaid. Two respondents reported unplanned pregnancy as a consequence of unaffordable birth control. D.F. is a 40-year-old woman with a prior history of unintended pregnancy after age 35. She states:

To be honest, $40 a month for contraception is impractical, I think immoral because most people can't afford that. And that's the insurance price. That's a co-pay. So goodness knows how much these drugs are actually charged for at the pharmacies at full price. They don't cost that much.

M.G., a 35-year-old woman with a prior history of unintended pregnancy after age 35, talks about the barriers she faced when paying for birth control: "I pay for the birth control stuff. When I got cut from my job I couldn't pay for the [monthly injectable method]. I made sure [my boyfriend] withdrew or used a condom. I got pregnant even though he withdrew." M.G. paid out of pocket for birth control; cost limited her ability to use her preferred choice of an effective birth control method and she resorted to less effective methods, resulting in an unintended pregnancy.

## Discussion

Understanding factors that relate to contraceptive use in women aged 35 and over may assist health care workers to provide more effective contraceptive counseling. Some factors discouraging contraception use are not unique and have been reported in other populations. For example, pregnancy ambivalence, low socioeconomic status, partnership status, attitudes towards various methods, method satisfaction, and contraceptive costs are some of the factors shown to influence a woman's use of a contraceptive method[5,19,20]. Other findings may be unique to older women, particularly when contraception is used to treat a chronic medical condition or avoid potential obstetrical complications. These findings suggest that health care providers should consider a life span approach to contraception counseling; contraception options and side effect profiles should be reviewed even for older women to ensure that correct information has been conveyed.

Most respondents expressed concerns about how contraception affected their health. Some respondents, after reassurance and counseling from their medical providers, chose a hormonal method because they understood it treated their current medical condition or provided other health benefits. None named additional important health benefits of hormonal contraceptives, such as the prevention of osteoporosis, ovarian cancer and endometrial cancer[21,22]. The same was true for the IUC. The majority of respondents discussed negative experiences that others had with the IUC, not taking into account that newer IUCs are now available. Misperceptions precluded respondents from considering potentially good methods. Such findings imply that accurate information about contraception needs to be communicated across the reproductive life span. Widespread public dissemination of information could help improve contraception use, particularly for those who have less access to the medical system.

Although the interview did not specifically ask about the advantages and disadvantages of periodic abstinence, many respondents mentioned it as a method that they were using or had used. Most applauded this method for its naturalness and freedom from chemicals. Although some unexpectedly conceived while using this method, discourse in its favor may imply that women in their late reproductive years are looking for a contraceptive method that does not require ingestion or injection of steroids, does not interrupt sexual intercourse and maintains normal menstruation.

Our study findings are subject to several limitations. First, respondents who agreed to participate probably differed in attitudes towards contraception than those who did not. Additionally, due to our small sample size, we were not able to adequately comment on how socioeconomic status may of affected contraception use. Third, our findings are not necessarily generalizable to other parts of the U.S.

## Conclusion

Understanding factors that influence contraception use and selection in women aged 35 and over at risk of unintended pregnancy is a critical step to improve contraception use in this population. Public health messages and health providers' care can help women in this age group by reviewing their fertility risks, as well as all contraceptive methods and their associated side effects. The impact of such interventions on unintended pregnancy rates in this age group should be tested in other areas of evidence-based medicine.

## List of abbreviations

(NSFG): National Survey of Family Growth; (IRB): Institutional Review Board; (IUC): intrauterine contraception; (FDA): Federal Drug Administration; (LUS): Levonorgestrel Uterine System; (PID): pelvic inflammatory disease; (DMPA): depot medroxyprogesterone; (SES): socioeconomic status.

## Competing interests

The authors declare that they have no competing interests.

## Authors' contributions

EMG was involved in data collection, study design, data analysis and interpretation, and drafting and writing of the manuscript. NPC was involved in study design, data analysis and interpretation, and drafting of the manuscript. SF participated in the data analysis and interpretation, and with drafting of the manuscript. KF was involved in study design and drafting of the manuscript. AD was involved in drafting of the manuscript. All authors read and approved the manuscript.

## Pre-publication history

The pre-publication history for this paper can be accessed here:

http://www.biomedcentral.com/1472-6874/11/5/prepub
